# Caloric restriction prevents the development of airway hyperresponsiveness in mice on a high fat diet

**DOI:** 10.1038/s41598-018-36651-2

**Published:** 2019-01-22

**Authors:** Haris Younas, Marcela Vieira, Chenjuan Gu, Rachel Lee, Mi-kyung Shin, Slava Berger, Jeff Loube, Andrew Nelson, Shannon Bevans-Fonti, Qiong Zhong, Franco R. D’Alessio, Meredith C. McCormack, Nadia N Hansel, Wayne Mitzner, Vsevolod Y Polotsky

**Affiliations:** 1 Division of Pulmonary and Critical Care Medicine, Department of Medicine, Johns Hopkins University School of Medicine, Baltimore, MD USA; 2 Department of Environmental Health and Engineering, Johns Hopkins Bloomberg School of Public Health, Baltimore, MD USA

## Abstract

We have previously shown that high fat diet (HFD) for 2 weeks increases airway hyperresponsiveness (AHR) to methacholine challenge in C57BL/6J mice in association with an increase in IL-1β levels in lung tissue. We hypothesize that obesity increases AHR via the IL-1β mechanism, which can be prevented by caloric restriction and IL-1β blockade. In this study, we fed C57BL/6J mice for 8 weeks with several hypercaloric diets, including HFD, HFD supplemented with fructose, high trans-fat diet (HTFD) supplemented with fructose, either *ad libitum* or restricting their food intake to match body weight to the mice on a chow diet (CD). We also assessed the effect of the IL-1β receptor blocker anakinra. All mice showed the same total respiratory resistance at baseline. All obese mice showed higher AHR at 30 mg/ml of methacholine compared to CD and food restricted groups, regardless of the diet. Obese mice showed significant increases in lung IL-1 β mRNA expression, but not the protein, compared to CD and food restricted mice. Anakinra abolished an increase in AHR. We conclude that obesity leads to the airway hyperresponsiveness preventable by caloric restriction and IL-1β blockade.

## Introduction

The prevalence of obesity is increasing worldwide. The epidemic of obesity can be attributed to increased consumption of high caloric food, sedentary life style and genetic factors. Obesity has been linked to multiple comorbidities including insulin resistance and type 2 diabetes, cardiovascular disorders, cancer and systemic low-grade inflammation^1^.

Asthma is a chronic inflammation of the bronchi leading to airway hyperresponsiveness (AHR), the major functional outcome of this disease. The relationship between obesity and asthma is well established^2–4^. Obese asthma poses a significant public health problem due to poor understanding of the pathogenesis and a lack of effective treatment. In the mouse model, IL-17, IL-1β and the NLRP3 inflammasome have been implicated in the pathogenesis of obesity-induced airway hyperresponsiveness^5^. Additionally, a high fat diet (HFD) has been linked to asthma, independent of obesity, although the underlying mechanism is not clear. Moreover, weight loss by caloric restriction improved disease control and quality of life in asthmatics^6^, but mechanisms are also insufficiently understood.

We have previously shown that HFD leads to AHR in mice after 2 weeks of feeding in association with IL-1 β upregulation in lung tissue, whereas other pro-inflammatory cytokines were unchanged^7^. However, it was unclear whether a small weight gain or dietary fat led to hyperresponsiveness and whether IL-1β played a causal role. In addition, emerging epidemiology literature suggests that a diet high in fructose is associated with asthma^8^. We hypothesized that obesity rather than a diet leads to AHR via the IL-1β mechanism and that caloric restriction on the same diet will abolish both AHR and pulmonary inflammation. We investigated our hypothesis in two experiments. In the first experiment, C57BL/6J mice were fed with several hypercaloric diets including HFD, HFD supplemented with 30% fructose added to drinking water, and high trans-fat diet (HTFD) supplemented with 30% fructose added to drinking water for 8 weeks, either *ad libitum* [HFD(O), HFD + HFr(O), and HTFD + HFr(O) groups, respectively] or food restricted to match their weight to the control group on a chow diet [HFD(R), HFD + HFr(R), and HTFD + HFr(R) groups, respectively], and subsequently AHR and pulmonary inflammation were measured. In the second experiment, C57BL/6J mice were fed with a HFD *ad libitum* for 8 weeks and treated with an IL-1β receptor blocker or placebo during the last 2 weeks of the experiment followed by the same measurements.

## Results

**Figure 1:**
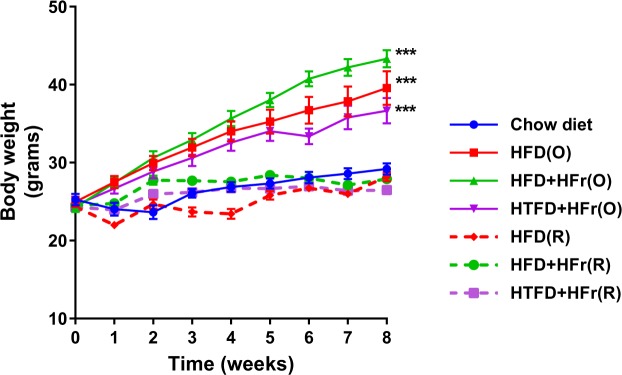
Weight trajectory of chow diet (CD), HFD (high fat diet), HFD + high fructose, HTFD + high fructose *ad libitum* (O) and restricted (R) groups over the period of 8 weeks. ^*,**,***^Denote that the final weight was significantly different as compared to the chow diet (CD), ^*^p < 0.05, ^**^p < 0.01, ^***^p < 0.001.

In the first experiment, mice in all groups gained significant amount of weight over the period of 8 weeks as compared to their initial weight. As expected, the hypercoloric *ad libitum* groups gained more weight compared to the chow (CD) group and the restricted HFD, HFD + HFr, HTFD + HFr groups (p < 0.001, Table 1). There was no weight difference between HFD(R), HFD + HFr(R), HTFD + + HFr(R) and CD group across the 8-week time course (Fig. 1). There was no difference in lung volumes measured by water displacement between the groups (Table 2).

**Table 1:** Basic characteristics, and plasma metabolic parameters in regular chow diet, HFD, HFD + high fructose, HTFD + high fructose *ad libitum* (O) and restricted (R) groups.

	CD	Obese			Food Restricted		
		HFD (O)	HFD + HFr (O)	HTFD + HFr (O)	HFD (R)	HFD + HFr (R)	HTFD + HFr (R)
Number of mice (n)	16	8	8	8	8	8	8
Age (weeks)	8	8	8	8	8	8	8
Initial weight (g)	25.26 ± 0.74	24.98 ± 0.67	24.26 ± 0.58	24.54 ± 0.60	24.35 ± 0.51	24.48 ± 0.30	24.29 ± 0.39
Final weight (g)	29.19 ± 0.71	39.56 ± 2.15^***^	43.31 ± 1.11^***^	36.65 ± 1.64^***^	28.19 ± 0.59	27.9 ± 0.50	26.49 ± 0.38^***^
Daily food intake (g/mouse)	3.12 ± 0.41	2.36 ± 0.30	2.47 ± 0.21	2.49 ± 0.21	1.64 ± 0.07	1.72 ± 0.02	1.71 ± 0.02
Daily food intake (KJ/mouse)	39.17 ± 5.20	52.26 ± 8.31	54.74 ± 4.71	46.69 ± 3.97	36.58 ± 3.37	38.06 ± 0.51	32.14 ± 0.43
Blood glucose (mg/dl)	139.4 ± 3.9	179.6 ± 16.8^*^	211.1 ± 9.8^***^	169.4 ± 7.5	127.0 ± 8.0	136.5 ± 8.1	100.9 ± 5.0^*^
Serum insulin (ng/ml)	0.49 ± 0.06	2.26 ± 0.73^*^	2.90 ± 0.55^***^	1.86 ± 0.56	0.22 ± 0.02	0.31 ± 0.06	0.24 ± 0.02
Serum leptin (ng/ml)	5.89 ± 0.85	33.22 ± 10.56^***^	45.72 ± 7.40^***^	10.92 ± 2.04	6.17 ± 1.29	3.51 ± 0.73	3.13 ± 0.99
Serum Adiponectin (μg/ml)	16.76 ± 2.81	12.07 ± 1.01	11.17 ± 0.78	9.60 ± 1.37	11.59 ± 0.94	11.40 ± 0.44	13.12 ± 0.61
Serum Triglyceride (mg/dl)	85.90 ± 9.50	85.44 ± 10.3	ND	ND	54.68 ± 6.94^**^	ND	ND
Serum FFA (mmol/l)	0.14 ± 0.03	0.15 ± 0.03	0.12 ± 0.04	0.10 ± 0.01	0.23 ± 0.05	0.30 ± 0.05^*^	0.41 ± 0.05^***^

^*,**,***^Denote that these values were significantly different as compared to the chow diet (CD) group. ^*^p < 0.05, ^**^p < 0.01, ^***^p < 0.001. ND, not done.

**Table 2:** Lung parameters and bronchoalveolar lavage (BAL) data in regular chow diet, HFD, HFD + high fructose, HTFD + high fructose *ad libitum* (O) and restricted (R) groups.

		CD	Obese			Food Restricted		
			HFD (O)	HFD + HFr (O)	HTFD + HFr (O)	HFD (R)	HFD + HFr (R)	HTFD + HFr (R)
Number of mice (n)		16	8	8	8	8	8	8
Age (weeks)		8	8	8	8	8	8	8
Triglycerides in lung tissue (µg/mg)		4.91 ± 0.38	5.82 ± 0.74	8.65 ± 1.17^***^	4.98 ± 0.45	5.8 ± 0.43	3.44 ± 0.29	5.15 ± 0.46
Free fatty acids in lung tissue (umol/mg)		0.022 ± 0.002	0.025 ± 0.003	0.026 ± 0.003	0.022 ± 0.001	0.020 ± 0.002	0.022 ± 0.003	0.03 ± 0.003
Lung volume (ml)		0.23 ± 0.02	0.22 ± 0.01	0.21 ± 0.05	0.23 ± 0.01	0.22 ± 0.01	0.24 ± 0.01	0.23 ± 0.01
Interleukin-1β mRNA in epididymal fat (fold change)		1.00 ± 0.15	1.01 ± 0.16	ND	ND	ND	ND	ND
Interleukin-1β mRNA in inguinal fat (fold change)		1.00 ± 0.33	0.44 ± 0.17	ND	ND	ND	ND	ND
BAL cell count (cells/ml)		27703.1 ± 4571.1	58531.3 ± 6299.4	61406.3 ± 13597.3	87268.8 ± 20707.6^***^	20714.3 ± 3965.1	41666.7 ± 10389.3	22500 ± 3273.3
BAL differential (% of total)	Epithelial cells	2.3 ± 0.5	1.5 ± 0.2	1.3 ± 0.3	1.5 ± 0.5	2.5 ± 0.6	0.7 ± 0.3	3.5 ± 1.1
	Macrophages	97.0 ± 0.5	97.9 ± 0.3	98.1 ± 0.4	97.7 ± 0.7	96.7 ± 0.5	98.9 ± 0.4	96.0 ± 1.3
	Eosinophils	0	0	0	0	0	0	0
	Basophils	0	0	0	0	0	0	0
	Neutrophils	0.07 ± 0.05	0.11 ± 0.06	0.08 ± 0.05	0.49 ± 0.33	0.03 ± 0.03	0	0.1 ± 0.1

^***^Denote that these values were significantly different as compared to the chow diet (CD) group, p < 0.001. ND, not done.

**Figure 2:**
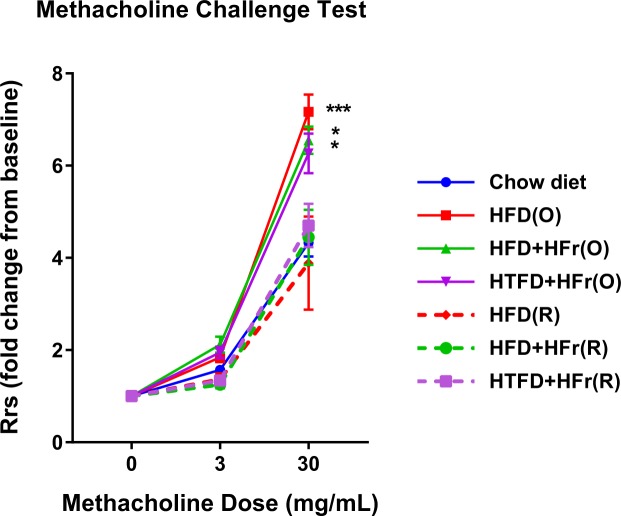
HFD, HFD + high fructose and HTFD + high fructose *ad libitum* (O) groups have increased total resistance of the respiratory system (Rrs) in response to methacholine as compared to chow diet group (^*^p < 0.05, ^***^p < 0.001). The Rrs values were normalized to baseline (no significant difference between groups at baseline). No difference in Rrs was observed between CD and caloric restricted groups.

Total respiratory resistance (Rrs) at baseline was identical in all seven groups, 1.00 ± 0.06 cmH_2_O.s/mL, 1.00 ± 0.02 cmH_2_O.s/mL, 1.00 ± 0.03 cmH_2_O.s/mL, 1.00 ± 0.02 cmH_2_O.s/mL, 1.00 ± 0.05 cmH_2_O.s/mL, 1.00 ± 0.04 cmH_2_O.s/mL, and 1.00 ± 0.02 cmH_2_O.s/mL in the CD, HFD(O), HFD + HFr(O), HTFD + HFr(O), HFD(R), HFD + HFr(R), HTFD + HFr(R) groups, respectively. AHR assessed by methacholine challenge showed no difference between the groups at 3 mg/ml dose of methacholine. However, when the methacholine dose was increased to 30 mg/ml, HFD(O), HFD + HFr(O) and HTFD + HFr(O) group exhibited a robust increase in airway reactivity (Fig. 2). Specifically, at this dose of methacholine, HFD(O), HFD + HFr(O) and HTFD + HFr(O) mice showed 7.2 ± 0.4, 6.6 ± 0.3 and 6.3 ± 0.4 fold increase in AHR from baseline *vs* 4.3 ± 0.3, 3.9 ± 1.0, 4.4 ± 0.6, and 4.7 ± 0.5 fold in CD, HFD(R), HFD + HFr(R) and HTFD + HFr(R) groups, respectively (p < 0.05).

**Figure 3:**
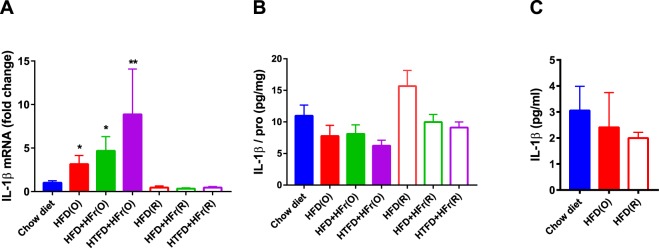
(**A**) Interleukin-1 beta (IL-1β) mRNA levels in lung tissue of chow diet (CD), HFD, HFD + high fructose, HTFD + high fructose *ad libitum* (O) and restricted (R) groups; (**B**) Interleukin-1 beta (IL-1β) protein levels in lung lysates of chow diet (CD), HFD, HFD + high fructose, HTFD + high fructose *ad libitum* (O) and restricted (R) groups. The results were adjusted by sample protein concentration. (**C**) IL-1β protein secretion to the media by adherent cells isolated from the lung single cell suspension in chow diet (CD), high fat diet *ad libitum* (O) and restricted (R) group. ^*,**^Denote that these values were significantly different as compared to the chow diet (CD), ^*^p < 0.05, ^**^p < 0.01.

Metabolic measurements showed that the HFD(O) and HFD + HFr(O) groups had higher fasting blood glucose levels compared to CD (p < 0.05, Table 1). Fasting blood glucose was lower in HTFD + HFr(R) group compared to CD group but not different between CD, HFD(R) and HFD + HFr(R) group (Table 1). Serum insulin and leptin levels were significantly higher in HFD(O), HFD + HFr(O) groups compared to the CD group. There was no difference in adiponectin levels across the groups (Table 1). Serum free fatty acids (FFAs) were higher in HFD + HFr(R) and HTFD + HFr(R) group than CD group. There was no difference in serum FFAs between HFD(O), HFD + HFr(O), HTFD + HFr(O) and CD groups. The HFD(R) group had lower triglycerides as compared to CD group and HFD (O) group. HFD(O), HFD + HFr(O) and HTFD + HFr(O) groups showed a greater than 2-fold increase in the bronchoalveolar lavage (BAL) cellularity with the predominance of macrophages (Table 2). Neither eosinophils nor basophils were observed in BAL, and the proportion of neutrophils were less than 1% in all groups. Lung triglycerides were higher in HFD + HFr(O) than CD group, but the values were similar between HFD(O), HTFD + HFr(O), caloric restricted groups and CD group (Table 2). There was no difference in lung FFAs across the groups (Table 2). Lung TNF-α and IL-6 were not significantly different between CD, HFD(O) and HFD(R) group (Suppl. Fig. 1). Such pro-inflammatory cytokines as IL-4, IL-5, IL-13, IL-17, IL-18, IL-21, IL-23 were not detected. There were a 3.1 ± 1.0-fold, 4.7 ± 1.6-fold and 8.7 ± 5.2-fold increases in IL-1 β mRNA levels in the lung tissue of HFD(O), HFD + HFr(O) and HTFD + HFr(O) mice compared to the CD group. In contrast, IL-1 β mRNA expression in lung tissue of the HFD(R), HFD + HFr(R), HTFD + HFr(R) groups were not elevated (Fig. 3A). However, IL-1 β protein levels in lung lysates were not significantly different across all the obese and caloric restricted groups, either (Fig. 3B). Flow cytometry also did not show any difference in cell-specific IL-1β production from pulmonary macrophages, neutrophils or lymphocytes between the groups (Suppl. Fig. 2). IL-1 β protein secretion by pulmonary macrophages was similarly not different between the CD, HFD(O) and HFD(R) groups (Fig. 3C). IL-1β mRNA levels in epididymal fat and inguinal fat tissues were not increased in mice fed on HFD *ad libitum* as compared to CD group (Table 2).

**Figure 4:**
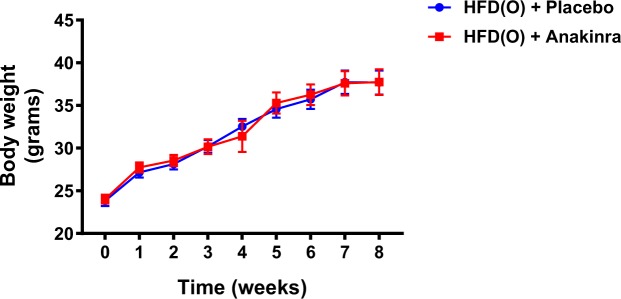
Weight trajectory of HFD *ad libitum* + placebo and HFD *ad libitum* + anakinra group over the period of 8 weeks.

**Figure 5:**
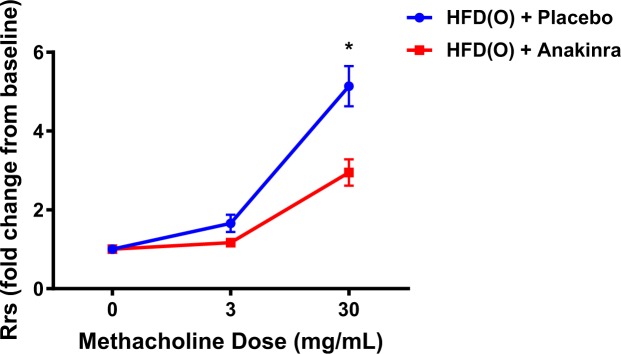
Anakinra injection decreased total resistance of the respiratory system (Rrs) in response to methacholine in HFD induced obese mice. The Rrs values were normalized to baseline (no significant difference between groups at baseline). ^*^Denotes p < 0.05.

In the second experiment we examined the effect of an IL-1β blocker anakinra on metabolic parameters and airway responsiveness in mice fed HFD. The placebo and anakinra groups gained similar amount of weight after feeding with HFD for 8 weeks (Fig. 4). Serum insulin levels were lower in the anakinra group compared to the placebo group (Table 3). There was no significant difference in fasting glucose, leptin, adiponectin, FFAs or triglyceride levels between two groups (Table 3). Anakinra had no effect on Rrs at baseline, 0.65 ± 0.03 cmH_2_O.s/mL *vs* 0.67 ± 0.03 cmH_2_O.s/mL in the placebo group. There was no difference between the groups at 3 mg/ml of methacholine. However, at 30 mg/mL of methacholine the AHR in the anakinra group was significantly lower compared to the placebo group (2.9 ± 0.9 *vs* 5.1 ± 1.4 respectively, p = 0.01) (Fig. 5). In fact, the AHR in obese mice treated with anakinra was identical to the lean CD mice and the HFDR mice from Experiment 1. The proportion of lymphocytes in BAL was lower in the anakinra group compared to the placebo group. The lung volumes, serum and lung FFAs, triglycerides and pro-inflammatory cytokines, total BAL cell count and other than lymphocyte cell content were not different between placebo and anakinra groups (Table 4).

**Table 3:** Basic characteristics, and plasma metabolic parameters in HFD *ad libitum* + placebo and HFD *ad libitum* + anakinra group.

	HFD (O) + placebo	HFD (O) + anakinra
Number of mice (n)	8	8
Age (weeks)	6–8	6–8
Initial weight (g)	23.8 ± 0.6	24.0 ± 0.5
Final weight (g)	37.7 ± 1.4	37.7 ± 1.5
Daily food intake (g/mouse/day)	2.14 ± 0.53	2.16 ± 0.54
Daily food intake (KJ/mouse)	48.32 ± 11.96	48.78 ± 12.17
Blood glucose (mg/dl)	159.0 ± 5.8	135.3 ± 12.5
Serum insulin (ng/ml)	2.53 ± 0.44	1.12 ± 0.41^*^
Serum leptin (ng/ml)	21.5 ± 5.2	26.0 ± 12.4
Serum Adiponectin (μg/ml)	11.2 ± 1.1	11.2 ± 0.7
Serum TG (mg/dl)	106.5 ± 11.8	112.3 ± 14.4
Serum FFA (mmol/l)	0.17 ± 0.04	0.18 ± 0.05

^**^Denote that this value was significantly different between two groups, p < 0.05.

**Table 4:** Lung parameters and bronchoalveolar lavage (BAL) data in HFD *ad libitum* + placebo group and HFD *ad libitum* + anakinra group.

		HFD (O) + Placebo	HFD (O) + Anakinra
Number of mice (n)		8	8
Triglycerides in lung tissue (µg/mg)		6.16 ± 1.27	4.74 ± 0.64
Free fatty acids in lung tissue (umol/mg)		0.017 ± 0.002	0.013 ± 0.001
Lung volumes (ml)		0.19 ± 0.004	0.21 ± 0.004
BAL cell count (cells/mL)		39771.4 ± 8404.7	28342.9 ± 4548.5
BAL differential (% of total)	Epithelial cells	1.1 ± 0.1	1.6 ± 0.4
	Macrophages	94.9 ± 0.1	95.4 ± 0.6
	Eosinophils	0	0
	Basophils	0	0
	Neutrophils	0	0
	Lymphocytes	4.6 ± 0.2	3.1 ± 0.4^**^

^**^Denote that this value was significantly different p < 0.01.

## Discussion

The main finding of our study was that, in mice on a high fat hypercaloric diet, caloric restriction prevented the development of airway hyperresponsiveness and upregulation of IL-1β gene expression in lung parenchyma, regardless of the diet. Moreover, IL-1β receptor blockade also prevented and maybe even reversed the development of airway hyperresponsiveness in obese mice, despite persistent metabolic abnormalities.

Our study was designed to determine whether obesity *per se* or diet lead to asthma. A positive correlation between high fat diet intake and asthma has been previously observed^9^. Wood *et al*. suggests that the abundance of saturated fatty acids and lack of antioxidants in HFD can induce inflammation by activating toll-like receptors and hence stimulating the NF-κB inflammatory cascade^10^. HFD also induces the proliferation of invasive bacteria and eliminates the protective bacteria in the gut which can induce inflammation^11,12^. A high fructose diet has also been associated with asthma^8^. Our data demonstrated that two different types of HFD fed *ad libitum* induced pulmonary inflammation with increased macrophages in BAL, especially in the HTFD + HFr group. However, our food restriction protocol showed that food restricted mice did not develop AHR, despite being fed the same diet. These results lead to the conclusion that the airway hyperresponsiveness is a consequence of obesity rather than high fat or high fructose diets. We recently reported that HFD feeding for 2 weeks led to an increase in AHR of the similar magnitude as HFD feeding for 8 weeks, despite much more significant weight gain in a longer term experiment^7^. Taken together these data suggest that even mild obesity can lead to airway hyperresponsiveness.

Several physiological and immunological mechanisms have been implicated in the pathogenesis of obese asthma. Excessive adiposity can have a restrictive effect on the lung decreasing functional residual capacity and expiratory reserve volume^13^. Radial traction around the distal airway is decreased at low lung volume contributing to airway narrowing^14,15^. A reduction in initial airway caliber allows a greater increase in resistance for a given absolute reduction in smooth muscle shortening, which manifests as increased airway reactivity^16^. Obesity’s effect on lung and chest wall compliance also lessens the effectiveness of a bronchoprotective deep breath to dilate airways^17^. In addition to these mechanical effects, our current study highlights an even more important role of pulmonary inflammation in the pathogenesis of obesity-induced airway hyperresponsiveness in mice.

Our previous study showed that HFD feeding for two weeks increases AHR in association with increased IL-1 β gene expression in the lung and augmented IL-1 β secretion by pulmonary macrophages^7^. The current study showed that the expression of IL-1 β mRNA was increased in the mouse lungs after HFD feeding for 8 weeks and this increase was prevented by caloric restriction. The IL-1β receptor blockade also prevented both obesity-induced AHR and pulmonary inflammation, supporting the concept that up-regulation of IL-1β gene expression in the lung could be a mechanism linking obesity and asthma^5^. Given that obesity increases AHR early in the time course^7^ and that anakinra was administered only during last two weeks of the 8-week experiment, our data suggest that IL-1β receptor blockers not merely prevent, but also reverse obese asthma.

Mechanisms of obesity-induced up-regulation of IL-1β have been linked to the NLRP3 inflammasome, which was examined in detail by Umetsu *et al*.^5^. However, we did not detect an increase in adipose IL-1β in mice on a HFD. We found an increase in lung triglycerides only in the HFD + HFr (O) group, whereas the AHR was increased in all obese groups. Nevertheless, the lung is an important organ of triglyceride rich lipoprotein clearance^18^ and it is conceivable that particular species of fatty acids, e.g. long chain fatty acids, which were not measured specifically in our study, induced IL-1β expression in the lungs. IL-1β could increase bronchial reactivity via up-regulation of T_H_2/T_H_17 cells, which were associated with severe asthma phenotype in humans^19,20^.

Other potential mechanisms contributing to obesity-induced pulmonary inflammation and AHR are hyperleptinemia^21^, hyperglycemia^22^ and insulin resistance^23,24^, which were observed in obese mice on a HFD, but not in control chow fed mice or food restricted mice on a HFD^25^. Of note, the IL-1β receptor blocker anakinra has been shown to improve hyperglycemia and insulin secretion in type 2 diabetes^26^. In our study, anakinra lowered fasting serum insulin levels without a significant change in fasting glucose levels indicating increased insulin sensitivity. Our finding suggests that anakinra could contribute to improvement in AHR due to its off-target effects by improving glucose metabolism.

The most important novel finding of our study was that caloric restriction prevented the development of airway hyperresponsiveness. The data from several trials showed that weight loss in asthmatics through caloric restriction can lead to clinical improvement^6,27–29^. We agree and further propose that caloric restriction may be beneficial for asthma in obese individuals, possibly by suppressing the IL-1β response.

Our study had several limitations. *First*, we were unable to demonstrate an increase in IL-1β protein. We have previously shown that IL-1β secretion by pulmonary macrophages is dramatically increased after 2 weeks of high fat diet^7^. After 8 weeks of HFD and development of severe obesity, IL-1β mRNA levels remained elevated, but the IL-1β protein levels and secretion by pulmonary macrophages were no longer increased. These data may suggest that IL-1β peaked early in the time course of HFD – induced obesity. *Second*, there was an apparent discrepancy between the lack of increase in IL-1β protein levels and beneficial effects of the IL-1β receptor blocker on AHR. Besides possible fluctuations of IL-1β levels over the time course, anakinra could exert a therapeutic effect in the absence of an increase in IL-1β by blocking the receptor and downstream inflammation. It is also possible that anakinra had non-specific off-target beneficial effects, for instance by alleviating insulin resistance as discussed above. *Third*, other than IL-1β mechanisms by which obesity may increase AHR were not addressed. Recent literature suggest that long chain fatty acids may induce inflammation by altering macrophage lipid metabolism indirectly via the toll receptor 4^30^. *Finally*, we did not investigate sex differences by including female mice.

## Conclusions and Implications

Diet induced obesity increases airway hyperresponsiveness and the effects of obesity are preventable by caloric restriction and IL-1β blockade. Taken together our data suggest that caloric restriction should be used for prevention of obese asthma and that IL-1β blockade may be considered as an adjunct therapy.

## Methods

### Animals and study design

The study was approved by the Institutional Animal Care and Use Committee of the Johns Hopkins University Animal Use and Care Committee (Protocol # MO15M257) and complied with the American Physiological Society Guidelines for Animal Studies. The study consisted of two experiments. In total, 80 C57BL/6J male mice (Jackson Labs Bar Harbor, MA), 6–8 weeks old) were used in the study. Mice were housed in 4 per cage, in a temperature and humidity-controlled room with a 12/12 light/dark cycle (9 am–9 pm lights on) with access to water at all time. 64 C57BL/6J male mice were used in the first experiment. Mice were divided in 7 groups, Control (CD) (n = 16), high fat diet *ad libitum* group [HFD(O)](n = 8), high fat diet + high fructose *ad libitum* group [(HFD + HFr)(O)](n = 8), high trans-fat diet + high fructose *ad libitum* group [(HTFD + HFr)(O)](n = 8), and the restricted HFD [HFD(R)](n = 8), HFD + HFr [(HFD + HFr)(R)](n = 8) and HTFD + HFr [(HTFD + HFr)(R)] groups. CD group was fed with chow diet (3.0 kcal/g, 4.4% fat, 13% kcal from fat) *ad libitum*. HFD(O) group was fed with high fat diet (TD 03584, Teklad WI, 5.4 kcal/g, 35.2% fat, 58.4% kcal from fat,) *ad libitum* for 8 weeks. (HFD + HFr)(O) group was fed with high fat diet (TD 03584) and fructose (30% by wt) *ad libitum* for 8 weeks. (HTFD + HFr)(R) group was fed with high trans-fat diet (Research diets, D09100301, 4.49 kcal/g, 19.9% fat, 40% kcal from fat] and fructose (30% by wt) *ad libitum* for 8 weeks. In caloric-restricted groups, mice were provided with the same HFD, HTFD and fructose but the amount of food was restricted to match body weight to the CD group. The composition of HFD has been described previously^7^. Diets were refrigerated at 4–8 °C before it was added to the cages. 16 C57BL/6J male mice were used in the second experiment. The second experiment consisted of 2 groups, placebo group (n = 8) and anakinra group (n = 8). Anakinra was a gift from Sobi (Stockholm, Sweden). Both groups were fed with HFD *ad libitum* for 8 weeks. During last 14 days of the experiment, the anakinra group was injected subcutaneously at 50 mg/kg in 250 µl of saline daily and placebo group was injected subcutaneously with 250 µl of saline daily.

### Physiological measurements

Mice were anesthetized with ketamine/xylazine i.p., tracheostomized and the total respiratory resistance (Rrs) was measured by forced oscillation technique (Flexivent, SCIREQ Québec, Canada) at baseline and after methacholine aerosol challenge at 3 and 30 mg/mL as described^7,31^. Blood was collected from the aorta, BAL was performed with 2 × 0.8 mL of sterile phosphate-buffered saline (PBS) through a tracheal cannula. The thorax was opened, and the right lung was tied off, dissected free and immediately frozen in liquid nitrogen and stored at −80 °C. Inguinal fat and epididymal fat tissue were collected, immediately frozen in liquid nitrogen and stored at −80 °C. The remaining left lung was inflated with formalin at 26 cmH_2_O pressure for 20 min, tied off and placed inflated in formalin for 2 days. Left lung volumes were measured by fluid displacement method^32^.

### Plasma, lung and adipose tissue analysis

Triglycerides and free fatty acids (FFA) were measured in lung homogenates and plasma with kits from Wako Inc (Richmond, VA). Plasma insulin, adiponectin and leptin were measured with kits from Alpco Diagnostics (Salem, NH), Millipore (Billerica, MA) and Abcam (Cambridge, MA), respectively. Blood glucose was measured following a 4-h fast by tail-snip technique using a handheld glucometer (ACCUCHECK Aviva Plus, Roche, Indianapolis, IN). IL-1β protein levels in lung lysates were measured with an ELISA kit (R&D systems). Total RNA was extracted from lung, inguinal fat and epididymal fat tissue with a Trizol reagent (Life Technologies, Rockville, MD). cDNA was produced from total RNA using Advantage RT for PCR kit from Clontech (Palo Alto, CA). Real time PCR was performed for the cytokine panel, including interleukins (IL) 1β, 4, 5, 6, 10, 13, 17, IL-18, TNF-α, IL-21, and IL-23 with premade primers from Invitrogen (Carlsbad, CA) and Taqman probes from Applied Biosystems (Foster City, CA) using 18S as a housekeeping gene. Custom made 18S primers were forward 5′-CTCTTTCGAGGCCCTGTAATTGT-3′, reverse, 5′-AACTGCAGCAACTTTAATATACGCTATT-3′ and the probe 6FAM-AGTCCACTTTAAATCCTT. Target mRNA level was normalized to 18 s rRNA, using the formula: Target/18 s = 2Ct(18 s)–Ct(target).

### Cytokine secretion and flow cytometry

In a subset of mice left lungs were harvested, minced and placed in gentle MACS Dissociator (Miltenyi Biotec), and digested using Collagenase type 1 (Worthington) and DNase I (Sigma Aldrich, St. Louis, MO) for 10 minutes at 37 °C. The lung digests were passed through a 70-μm nylon cell strainer (Becton Dickinson, Franklin Lakes, NJ), and erythrocytes were subsequently lysed using RBC Lysis Buffer (eBioscience, San Diego, CA). The cells were counted, and cells viability was assessed by Trypan Blue staining. Then, 2 × 10^6^ of viable cells were seeded in 96 well plates in the presence of DMEM + 10% FBS + Pen/Strep 1:100 medium. Two hours later, non-adherent cells were removed and 100 ul of the medium were added to the attached cells. The cells were incubated for 24 hours at 37 °C, the media was collected, centrifuged to remove cells and debris, and IL-1β secretion was measured with an ELISA kit (R&D systems). Four hours before end of the *ex vivo* culture, cells were stimulated with Phorbol myristate acetate (PMA, 40 ng/ml)) and ionomycin (500 ng/ml) in the presence of Golgistop (BD Biosciences). For flow cytometry, cells were washed with FACS buffer (PBS + 0.5% BSA) and incubated with PE-Cy CD64 Ab. Then, non-specific staining of Fcγ III/II receptors was blocked with Fc Block-2.4G2 (BD Biosciences — Pharmingen) Ab. The following Abs (BD Biosciences — Pharmingen) were used for cell phenotyping: PerCp Cy 5.5-conjugated anti-CD11c, PE-CF594-conjugated anti-CD11b, APC-Cy7-conjugated anti-MHCII, BV421-conjugated anti-SigF, BV605-conjugated anti-Ly6c, BV510-conjugated anti-Ly6g, BV395-conjugated anti-CD4 and BV737-conjugated anti-CD8 and respective isotype Abs. Cells were subsequently fixated and permeabilized and stained for IL-1β (Thermo Fisher). Lymphocytes, monocytes, neutrophils, alveolar and interstitial macrophages were gated with characteristic low forward scatter/side scatter, using a FACSAria instrument and FACSDiva for data acquisition (Becton Dickinson) and Flowjo for analysis (Tree Star Inc.) as previously described^33^.

### Statistical analysis

The statistical analysis was done using STATA version 12. All values were reported as means ± SEM. All the data in the study was checked for normality with Jarque-Bera test. Statistical analysis of normally distributed variables was determined by student t-test or one-way analysis of variance test (ANOVA) with repeated measures when appropriate. Non-normally distributed values were analyzed by Kruskal-Wallis rank test. A p-value of <0.05 was considered significant.

## Electronic supplementary material

Supplementary material
