# Supplementary material for: Caloric restriction prevents the development of airway hyperresponsiveness in mice on a high fat diet
Source: Sci Rep. 2019 Jan 22;9:279. doi: 10.1038/s41598-018-36651-2 (PMC6342916; doi:10.1038/s41598-018-36651-2)
Supplement: Supplementary file 1 — Supplementary material [file 41598_2018_36651_MOESM1_ESM.docx]

**Caloric restriction prevents the development of airway hyperresponsiveness in mice on a high fat diet**

Haris Younas^1^, Marcela Vieira^1^, Chenjuan Gu^1^, Rachel Lee^1^, Mi-kyung Shin^1^, Slava Berger^1^, Jeff Loube^2^, Andrew Nelson^2^, Shannon Bevans-Fonti^1^, Qiong Zhong^1^, Franco R D’Alessio^1^, Meredith C. McCormack^1^, Nadia N Hansel^1^, Wayne Mitzner^2^, and Vsevolod Y Polotsky^1^*

^1^Division of Pulmonary and Critical Care Medicine, Department of Medicine, Johns Hopkins University School of Medicine;

^2^Department of Environmental Health and Engineering, Johns Hopkins Bloomberg School of Public Health, Baltimore, MD

Corresponding author:

Vsevolod (Seva) Y. Polotsky, MD, PhD

Professor of Medicine

Division of Pulmonary and Critical Care Medicine

Department of Medicine, The Johns Hopkins University School of Medicine

5501 Hopkins Bayview Circle, Johns Hopkins Asthma and Allergy Center, Rm 4B65

Baltimore, MD 21224

Tel.: 410-550-6386

Fax: 410-550-2612

e-mail: vpolots1@jhmi.edu

website: https://www.polotskylab.net/

**Supplementary figure legends**

**Figure 1**: Tumor necrosis factor α (TNF-α) and interleukin 6 (IL-6) mRNA levels in lung tissue showed no difference between the chow diet group, high fat diet obese group and high fat diet restricted group.

**Figure 2:** The effect of high fat diet (HFD) feeding on IL-1β positive leukocyte populations in the mouse lungs. CD4+, CD8+ lymphocytes, interstitial macrophages (CD64^+^CD11b^+^CD11c^+^MHC II^+^), monocytes (CD11b^+^Ly6C^+^Ly6G^-^), and alveolar macrophages (CD11c^+^SiglecF^+^CD64^+^MHC II^+^) and neutrophils (CD11b^+^Ly6C^-^Ly6G^+^) were identified by flow cytometry according to protocol described by Misharin et al. (ref.27). HFDO, high fat diet obese (fed *ad libitum*); CD, chow diet; HFDR, high fat diet food restricted; S, stimulated cells; NS, non-stimulated cells.

**Supplementary Figure 1:**

**Supplementary Figure 2:**
